# Biocompatible Natural Polymer-Based Amorphous Solid Dispersion System Improving Drug Physicochemical Properties, Stability, and Efficacy

**DOI:** 10.3390/polym17152059

**Published:** 2025-07-28

**Authors:** Arif Budiman, Helen Ivana, Kelly Angeline Huang, Stella Aurelia Huang, Mazaya Salwa Nadhira, Agus Rusdin, Diah Lia Aulifa

**Affiliations:** 1 Department of Pharmaceutics and Pharmaceutical Technology, Faculty of Pharmacy, Universitas Padjadjaran, Jl. Raya Bandung-Sumedang Km. 21, Bandung 45363, Indonesia; kelly22001@mail.unpad.ac.id (K.A.H.); stella22001@mail.unpad.ac.id (S.A.H.); 2 Department of Pharmaceutical Analysis and Medicinal Chemistry, Faculty of Pharmacy, Universitas Padjadjaran, Jl. Raya Bandung-Sumedang Km. 21, Bandung 45363, Indonesia; helen22001@mail.unpad.ac.id (H.I.); mazaya22001@mail.unpad.ac.id (M.S.N.); agusrusdin@gmail.com (A.R.); diah.lia@unpad.ac.id (D.L.A.)

**Keywords:** amorphous solid dispersion, natural polymer, solubility, dissolution, biocompatibility

## Abstract

Poor aqueous solubility still disqualifies many promising drug candidates at late stages of development. Amorphous solid dispersion (ASD) technology solves this limitation by trapping the active pharmaceutical ingredient (API) in a high-energy, non-crystalline form, yet most marketed ASDs rely on synthetic carriers such as polyvinylpyrrolidone (PVP) and hydroxypropyl methylcellulose (HPMC), which raise concerns about long-term biocompatibility, residual solvent load, and sustainability. This study summarizes the emergence of natural polymer-based ASDs (NP-ASDs), along with the bond mechanism reactions through which these natural polymers enhance drug performance. As a result, NP-ASDs exhibit improved physical stability and significantly enhance the dissolution rate of poorly soluble drugs. The structural features of natural polymers play a critical role in stabilizing the amorphous state and modulating drug release profiles. These findings support the growing potential of NP-ASDs as sustainable and biocompatible alternatives to synthetic carriers in pharmaceutical development.

## 1. Introduction

The development of effective oral pharmaceutical formulations remains a formidable challenge, particularly for poorly water-soluble drugs, which represent nearly 70–90% of newly discovered active pharmaceutical ingredients (APIs). Low aqueous solubility often correlates with poor dissolution profiles, limited membrane permeability, and ultimately, reduced oral bioavailability, thereby compromising therapeutic efficacy [1,2,3]. These physicochemical limitations hinder optimal drug absorption and necessitate the use of high doses, potentially increasing the risk of adverse effects and patient non-compliance [4]. Hence, improving drug solubility has become a central goal in modern formulation science.

In response to this issue, numerous solubility enhancement strategies have been developed, including micronization [5], pH adjustment [6], salt formation [7], lipid-based formulations [8], cyclodextrin inclusion complexes [9], and solid dispersion systems [10]. Among these, amorphous solid dispersions (ASDs) have garnered significant attention for their ability to transform crystalline APIs into high-energy amorphous states that exhibit superior solubility and dissolution rates. These systems work primarily through molecular dispersion of the drug within a polymeric matrix, which enhances wettability and surface area and maintains the supersaturated state during gastrointestinal transit [11]. Consequently, ASDs represent a potent strategy to overcome solubility-limited absorption, making them a widely adopted platform in preclinical and clinical formulation pipelines.

However, despite their promise, conventional ASDs often rely on synthetic and semi-synthetic polymers such as polyvinylpyrrolidone (PVP) [12], polyethylene glycol (PEG) [13], or hydroxypropyl methylcellulose (HPMC) [14]. While these polymers exhibit acceptable functionality, they also present limitations, including potential toxicity, undesirable degradation byproducts, and limited biocompatibility, especially for long-term or implantable drug delivery systems [15]. In certain instances, synthetic excipients may elicit inflammatory responses or produce reactive intermediates that compromise patient safety. Additionally, the environmental burden associated with the production and disposal of synthetic polymers further illustrates the importance of safer, greener alternatives.

To address these deficiencies, the pharmaceutical field has turned its attention toward the use of natural polymers as the carrier matrix in ASD systems. These biopolymers—such as chitosan [16], alginate [17], hyaluronic acid [18], pectin [19], and starch [20]—are not only biodegradable and biocompatible but also offer intrinsic biological activities, mucoadhesive properties, and enzymatic responsiveness that can be tailored for targeted drug delivery [21,22]. Moreover, their derivation from renewable sources contributes to the sustainability of pharmaceutical manufacturing. Recent studies have demonstrated that natural polymers can effectively stabilize the amorphous form of various APIs, inhibit recrystallization, and significantly enhance dissolution rates [23]. Their inherent safety profiles make them especially suitable for chronic use, pediatric formulations, and sensitive therapeutic areas.

Despite the growing body of experimental research showcasing the success of natural polymer-based ASDs, there remains a notable lack of comprehensive review articles that consolidate this progress. The existing literature tends to focus on either individual polymers or generalized ASD concepts, without providing a focused evaluation of how natural polymers have been successfully applied to overcome the solubility barrier while simultaneously delivering a superior safety margin. This gap points to the need for a focused scholarly synthesis that not only maps the advancement of these natural polymeric systems but also elucidates their mechanistic superiority and formulation advantages over their synthetic counterparts. The application of chitosan oligosaccharide as a carrier for curcumin markedly improved its dissolution profile, elevating its solubility from 60.62 µg/mL to over 97 µg/mL, while sustaining supersaturation for 24 h, thereby illustrating enhanced bioavailability and improved physical stability [24]. The sodium alginate-based solid dispersion of indomethacin achieved a nearly complete drug release within five minutes, in contrast to a mere 5.3% release from the pure drug. This underscores alginate’s function in enhancing dissolution rates and storage stability, especially under accelerated settings [25]. These examples highlight the significant potential of natural polymers in improving therapeutic efficacy and providing resilient, biocompatible, and sustainable ASD systems.

Therefore, this review aims to systematically explore and critically assess the development of natural polymer-based ASD systems, highlighting their physicochemical mechanisms, solubility enhancement capabilities, improved biocompatibility, and safety profiles. By integrating detailed case studies and comparative analysis with synthetic polymers, this article seeks to provide a valuable reference for researchers and formulators seeking safer, more effective alternatives in drug delivery. Ultimately, this review contributes a much-needed perspective that bridges the current knowledge gap and offers strategic insights into the future of sustainable and biocompatible ASD technologies.

## 2. Methodology

**Figure 1:**
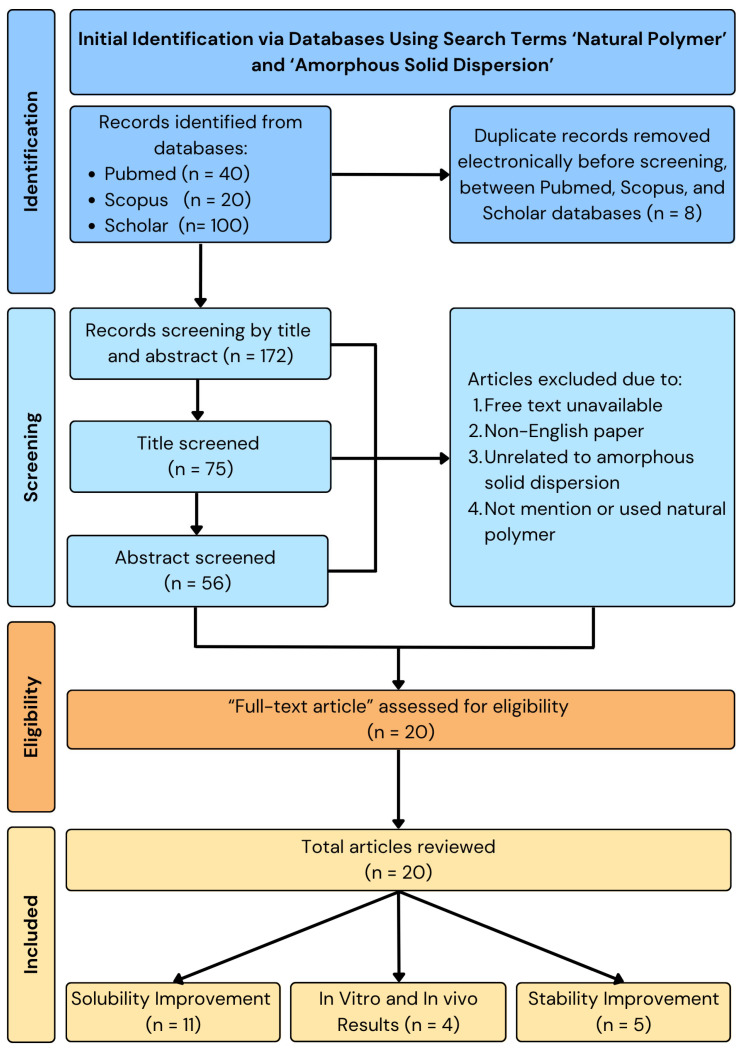
Methodology of natural polymer-based ASD.

This review uses a storytelling method to thoroughly examine and combine the latest developments in using natural polymers in ASD systems. Relevant literature was systematically retrieved from major scientific databases, including Clarivate Analytics, PubMed, Scopus, ScienceDirect, and Google Scholar. The search strategy employed a combination of keywords such as “natural polymer,” “amorphous solid dispersion,” “solubility enhancement,” “dissolution improvement,” “biodegradability,” “biocompatibility,” and “drug delivery systems,” covering publications from the years 2000 to 2024. Only peer-reviewed journal articles written in English were included. Priority was given to studies that specifically investigated the role of natural polymers as the primary carrier in ASD formulations. The exclusion criteria encompassed studies that focused solely on synthetic or semi-synthetic polymers, lacked experimental or formulation data, or did not provide measurable outcomes related to solubility, dissolution rate, or safety profiles. Data from the selected studies were extracted and synthesized thematically, emphasizing the physicochemical interactions, in vitro and in vivo performance, and comparative advantages over synthetic polymer-based systems. The narrative review format was deemed appropriate to accommodate the diverse formulation strategies and polymer types explored in the literature, allowing for a critical and integrative discussion of their mechanisms and therapeutic implications in improving drug solubility, stability, and safety. The flowchart of the methodology can be seen in Figure 1.

## 3. Natural Polymers

Natural polymers are molecules derived from biological sources such as plants, animals, and microorganisms. Natural polymers are believed to have better biocompatibility and biodegradability than synthetic polymers. Due to their similarity to tissues in terms of macromolecular structure, natural polymers offer the convenience of recognition from the biological system. Natural polymers are susceptible to breakdown by enzymes found in the body. However, it is feasible to influence how quickly this degradation happens by altering the polymer’s structure through techniques like chemical crosslinking or other modifications [26]. Natural polymers have emerged as promising candidates for the sustainable development of materials in areas ranging from food packaging and biomedicine to energy storage and electronics [27].

### 3.1. Types of Natural Polymer Sources

Natural polymers, encompassing polysaccharides (such as cellulose, chitosan, pullulan, hyaluronic acid, fucoidan, alginate, and agar) and proteins (including lysozyme and gelatin), are employed in the fabrication of composites, coatings, films, membranes, patches, nanosystems, and microneedles via eco-friendly methodologies, while catering to their principal application areas [27]. To elucidate the subject further, the table below (Table 1) delineates the diverse categories of natural polymers, accompanied by their defining function and representative examples.

### 3.2. Biodegradable and Biocompatible Properties of Natural Polymers

Biodegradable polymers are a class of materials that break down into harmless substances over time. This characteristic makes them highly attractive for use in drug delivery systems, as they can be designed to release their payload at a controlled rate and then disappear, minimizing the risk of side effects [44]. Among the benefits are the similarity to the host tissue, the capacity to interface with biological systems, the metabolic compatibility, the nontoxicity and low inflammatory responses, the enzyme-degradable nature, and the utilization of their breakdown products in cellular metabolism. However, due to their intricate structure and temperature sensitivity, natural polymers undergo destruction before they reach their melting point [16]. One acknowledged disadvantage of these polymers is the potential for disease transmission to humans from other species as a result of the production of various natural polymers using plant and animal resources [45].

Several key aspects influence the performance of biodegradable polymers in targeted drug delivery. In situ administration of formulations refers to the ability to deliver the drug directly to the target site. It ensures localized delivery of the drug, enhancing therapeutic efficacy while reducing systemic side effects. On-demand delivery of the molecularly targeted agent means that the drug is released only when needed, triggered by a specific signal or event. Such delivery can be accomplished through different mechanisms, including polymers that respond to changes in pH or the presence of specific enzymes. A targeted agent can be combined with radiotherapy and immunotherapy. Biodegradable polymers can be used to deliver drugs in conjunction with other treatments, such as radiotherapy or immunotherapy [46]. FDA-approved biodegradable polymers provide a dependable basis for the creation of biodegradable drug delivery systems. Clinical translation is accelerated by their approval, which guarantees safety, efficacy, and reproducibility. This kind of arrangement will help lower the possibility of issues during the product development process. Since natural polymers are essentially polysaccharides, they are biocompatible and harmless [47].

### 3.3. Advantages of Natural Polymers Compared to Synthetic Polymers

Natural polymers derived from marine sources have garnered significant interest due to their greater abundance and biological activity compared to polymers from other origins. A previous article suggests that natural polymers could be the first biodegradable biomaterials used in the therapeutic stage. Because of their bioactive qualities and improved cell–cell connections, they can improve the function of cells in biological systems. These natural polymers have been utilized for thousands of years. They consist of similar or varying repeating units. Typically, they are biodegradable and environmentally friendly. Synthetic polymers are widely used in construction, medicine, textiles, and packaging. A very broad and adaptable class of materials, synthetic polymers include, among other things, solubilizing agents, nanoparticulate formation, surface modification, drug carriers, diagnostic imaging agents, and implants. Many of these materials have been specifically used in drug delivery [15]. Natural polymers are less toxic and biodegradable, and they do not contain any synthetic chemicals, unlike synthetic polymers and their derivatives.

Proteins, nucleic acids, and polysaccharides are examples of natural polymers that are part of biological systems and carry out various vital tasks. Most biopolymeric films used in active food packaging are made from nanocelluloses or other film-forming polymeric matrices like pullulan, chitosan, and agar. Silva et al. found a variety of uses for natural polymers in innovative fields. Researchers created flexible electroconductive surfaces in electronics by combining copper nanowires with nanocellulose or poly-polystyrene sulfonate. For metal protection, chitosan coatings containing corrosion inhibitors were developed, and lanthanopolyoxometalates or corrole macrocycles were used to make functionalized chitosan films with luminous or imaging qualities. Biosorbent membranes derived from carboxylated or bacterial nanocellulose were successful in eliminating dyes and heavy metals such as mercury from water. These developments showcase the potential of natural polymers to offer a diverse array of technological and environmental solutions [27].

Natural polymers generally offer a better safety profile than synthetic ones due to their biocompatibility and biodegradability. A study on chitosan- and hyaluronic acid-based materials demonstrated minimal cytotoxicity, supporting their biomedical potential [48]. While natural polymers are often preferred for safety, thorough evaluation is essential, as processing methods and additives can influence their overall biocompatibility [15].

According to stability experiments, alginate increased the stability of solid dispersions irrespective of the drug’s current condition and produced a more stable result than an HPMCAS-based system. Alginate increases drug wettability and decreases particle agglomeration, greatly accelerating the dissolution rate of BCS II medications (lovastatin and indomethacin, for example). Compared to HPMC, alginate exhibits better stability under accelerated storage conditions. The amorphous-to-crystalline transition that takes place in HPMC-based systems considerably lowers dissolution, and alginate-based systems did not exhibit a noticeable rise in birefringence. On the other hand, microstructural phase separation causes relatively slight dissolution decreases in alginate-based systems, which have no effect on the effectiveness of pharmaceuticals. This suggests that the alginate-based system is more stable than the HPMCAS-based one [25].

Although polyvinylpyrrolidone (PVP) is generally considered safe for use in cosmetics, certain studies highlight potential disadvantages. A disadvantage of PVP involves its potential carcinogenic effects when implanted subcutaneously. According to studies, the implantation of particulate PVP in rats, mice, and rabbits resulted in the formation of localized tumor sarcomas at the site of implantation. While these findings do not indicate a systemic carcinogenic risk, they raise concerns about the safety of PVP in medical or cosmetic applications involving prolonged tissue exposure. The results suggest that while PVP is generally safe for topical use, its use in implanted or long-term exposure scenarios should be carefully evaluated to avoid potential health risks [49].

Chitosan offers numerous advantages across various fields, particularly in biomedicine, the food industry, and environmental applications. Its biodegradability and biocompatibility make it an environmentally friendly material that naturally decomposes without leaving harmful residues [48]. Additionally, it is safe for medical use, as it does not trigger toxic or allergic reactions. Chitosan also possesses antibacterial and antifungal properties, allowing it to inhibit the growth of harmful microorganisms, making it highly useful in food preservation and medical applications such as wound dressings and pharmaceuticals [50]. Another significant benefit of chitosan is its ability to absorb heavy metals, such as lead and mercury, from wastewater, making it a valuable component in water treatment and environmental remediation. In the food industry, it serves as a natural emulsifier and preservative, helping extend food’s shelf-life without the use of harmful chemicals [51]. Drug delivery systems widely study chitosan because it facilitates controlled drug release in the body, enhancing treatment effectiveness [52]. Furthermore, chitosan provides health benefits in dietary applications, as it can help lower cholesterol levels and bind fats in the body, making it a popular ingredient in dietary supplements and weight management products [44]. With its diverse applications and remarkable properties, chitosan continues to be an essential material in various industries, contributing to advancements in healthcare, food safety, and environmental sustainability [53].

Natural polymers offer several critical advantages that make them superior candidates for pharmaceutical applications, particularly in the development of ASD systems, as shown in Table 2. Unlike synthetic polymers such as poly(lactic-co-glycolic acid) (PLGA) and polylactic acid (PLA), which are often derived from petrochemical sources and may carry concerns regarding residual solvent content and long-term biocompatibility, natural polymers are typically processed in aqueous environments. This eliminates the need for organic solvents, reducing toxicity risks and making them more suitable for sensitive drug substances. Moreover, the renewable and biodegradable nature of natural polymers contributes to their environmental sustainability, with sources such as chitosan derived from shrimp shells and alginate extracted from brown algae being both abundant and eco-friendly. In terms of biological performance, natural polymers exhibit excellent biocompatibility and often possess inherent biological affinity for tissues and biomolecules. For example, chitosan demonstrates strong interactions with nucleic acids (DNA and RNA), while natural proteins such as collagen and fibrin support cell adhesion and tissue regeneration. Although some natural polymers may generate mildly acidic degradation products, their overall safety profile, minimal immunogenicity, and favorable interaction with biological systems make them highly attractive for drug delivery applications when compared to their synthetic counterparts, which may elicit inflammatory responses or accumulate due to incomplete biodegradation [54].

## 4. Amorphous Solid Dispersions

### 4.1. Basic Concept of ASDs

**Figure 2:**
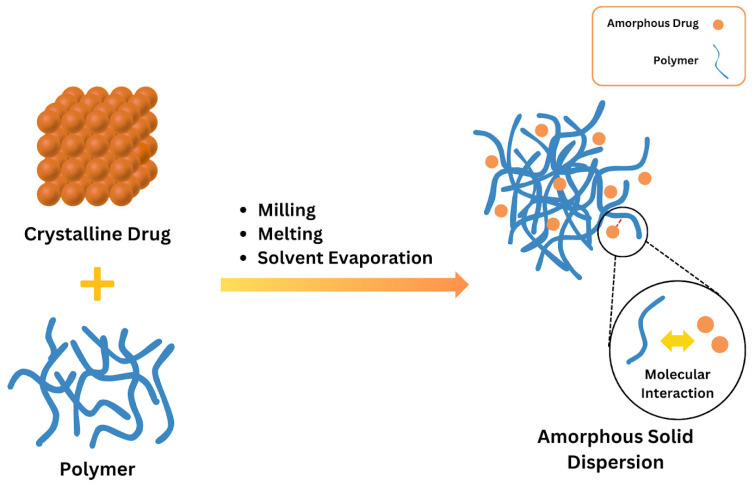
Basic concept of ASDs. Adapted from [62], Elsevier, 2023.

ASDs are characterized by the reduction in drug particle size to a molecular level, which solubilizes or co-dissolves the drug in the soluble carriers. They are molecular mixtures of poorly water-soluble drugs with hydrophilic carriers, which modulate the drug release profile. Since the medication is in its supersaturated condition as a result of forced solubilization in the hydrophilic carriers, they often offer higher wettability and dispersibility [56]. A polymer carrier stabilizes the crystalline medication in the ASD once it transitions into its amorphous state; this explanation can be seen in Figure 2, where the spheres represent single molecules or molecular clusters [54,56]. By reducing the movement of the drug’s molecules and increasing its glass transition temperature (Tg), the polymer carrier helps the drug dissolve better and stay stable in its solid form. The molecule may offer additional benefits when it is only available in its amorphous state. During production, shipping, or storage, this method stabilizes an amorphous product that is not chemically stable. Furthermore, when the neat amorphous molecule is insufficient, this method preserves supersaturation. The stability of an ASD is most likely caused by breaking intermolecular bonds in the drug’s crystal structure and forming a drug–polymer complex [56,57,58,59]. Disrupting intermolecular connections in the drug’s crystal structure and creating drug–polymer interactions are undoubtedly the causes of an ASD’s stability. The drug’s crystal structure affects how the polymer interacts with it, which influences the stability of the ASD. The thermodynamic and kinetic factors increase the bioavailability of the ASD [60,61].

### 4.2. Advanced Development of ASD Systems

To significantly enhance the bioavailability of insoluble drugs, the ASD method has lately been utilized as an effective formulation strategy. Reports indicate that 70 to 90 percent of all novel chemical entities (NCEs) or therapeutic compounds in development exhibit low water solubility, hence categorizing them as class II or class IV drugs under the Biopharmaceutics Classification System (BCS) [63]. The identified structure and functional groups during the drug discovery phase are responsible for this phenomenon of inadequate solubility. Saturation solubility and stability are the two fundamental elements of preformulation assessment, both of which are inherently affected by the properties of the active pharmaceutical ingredient (API) and any supplementary excipients utilized in the formulation. Recent decades have witnessed extensive research, primarily concentrating on the amorphous properties of APIs, the selection of suitable excipients, and the forecasting of the physical stability of ASDs [1].

High solubility can be achieved by combining widely used polymers with poorly soluble compounds. Choosing the right polymers is crucial to maximizing ASD performance. Recent studies have explored various polymers, which have shown promising results in stabilizing ASDs and enhancing drug solubility. Polymeric materials have numerous advantages and produce ASDs. By stopping crystallization, the polymers in ASDs can often make amorphous medications more stable. Furthermore, the presence of hydrophilic carriers makes the amorphous medicines more wettable. Additionally, by preventing drug precipitation in the gastrointestinal tract, the right polymeric carriers increase bioavailability [64].

One proposed explanation for the increased drug release with polymeric salts is that the polymeric carrier is pre-ionized, resulting in a heightened degree and rate of hydration. This enhances the liberation of polymer chains from the matrix and generates a more mobile gel layer, hence augmenting drug release. The absence of proton production during the hydration of polymeric salts necessitates the maintenance of a stable microenvironmental pH at the solid–water interface, which further enhances drug release when utilizing polymeric salts. While investigations on the physical stability of ASDs employing polymeric salts are ongoing, the application of polymeric salts may represent an effective formulation technique for developing ASDs with enhanced drug release [58]. Advanced characterization techniques, including differential scanning calorimetry (DSC) and dynamic mechanical analysis (DMA), are employed to evaluate the thermal characteristics and stability of ASDs at a molecular level [65].

The use of ASDs to improve the solubility and bioavailability of medications that are not very soluble in water has grown in popularity. ASDs are frequently added to pills to make oral administration easier. For amorphous medicines to have an inherent solubility advantage, disintegration and drug release from ASD tablets are therefore essential [66]. When a medicine is accessible in an amorphous form, ASDs can eventually increase its bioavailability. The choice of an appropriate polymer carrier aids in improving the drug’s solubility, dissolution rate, and physical stability in the solid form. A polymer carrier aids in the transformation of crystalline drugs into their amorphous forms and stabilizes ASDs by increasing the glass transition temperature (Tg) and decreasing molecular mobility [60].

### 4.3. Preparation Technique of ASDs

ASD systems can be prepared using solvent-based techniques such as freeze-drying, solvent evaporation, and spray-drying. To comply with the ICH Q3 (R5) requirements, these techniques necessitate meticulous solvent removal. They work well with polymers that have high melting points, but both the medication and the carrier must be sufficiently soluble [67]. Methanol, ethanol, and acetone are examples of common solvents. Alternatives such as supercritical/near-critical CO_2_ are being investigated, since they are safer and less harmful. The solvent approach is still useful at the laboratory level for problems like drug and polymer breakdown at high temperatures, despite its drawbacks [56].

**Figure 3:**
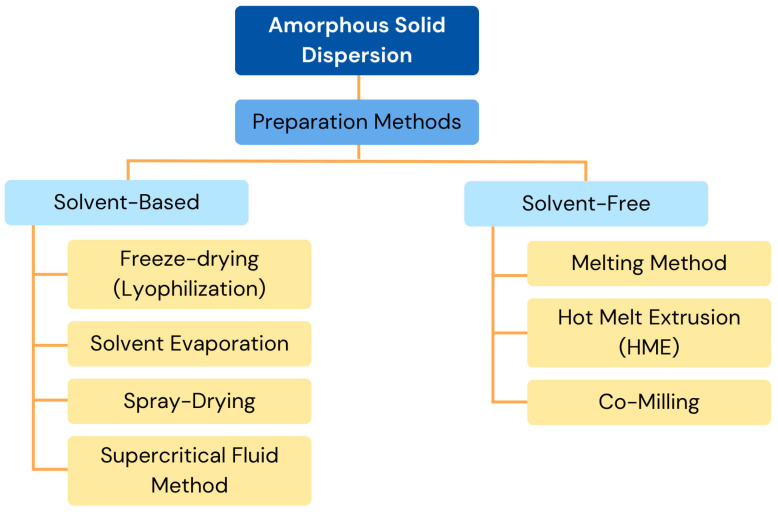
Preparation methods of ASDs. Adapted from [61,71], MDPI, 2023.

Various techniques prepare solid dispersions, each with unique benefits and difficulties (Figure 3). Although the technique takes a long time and is prone to drug recrystallization, rotary evaporation is perfect for small-scale production, since it avoids heat-induced deterioration. Because of its affordability, scalability, and capacity to guarantee molecular dispersion, spray-drying is frequently employed for large-scale production; nonetheless, the solvent composition may cause phase separation [68]. Although the technique takes a lot of time and may have problems with low-freezing-point solvents, freeze-drying, also known as lyophilization, is useful for thermolabile chemicals because it reduces thermal and aqueous stress [69]. The supercritical fluid method, usually using supercritical carbon dioxide, offers benefits for the environment, precise control of particles, and less heat damage; however, it requires a large upfront cost and careful handling of solvents [70].

When developing ASD systems, solvent-free techniques are quicker and have a better trapping efficiency than solvent-based techniques. Because they do not involve the use of organic solvents, these techniques are environmentally safe and make it simple to anticipate the drug concentration based on component ratios. The melting method, which offers solvent-free processing but runs the risk of thermal deterioration, depends on heating materials over the eutectic point for matrix integration. Even though this method only works for heat-stable chemicals, hot melt extrusion (HME) mixes medications and polymers through controlled extrusion, ensuring even distribution and the ability to produce continuously [56]. Through carefully blended drug–excipient mixes, co-milling, a method for reducing particle size, improves amorphization, solubility, and bioavailability; nevertheless, residual crystallinity may present stability issues. When combined, these methods solve different formulation problems and optimize drug administration efficacy.

## 5. Characterization and Evaluation of ASDs

**Figure 4:**
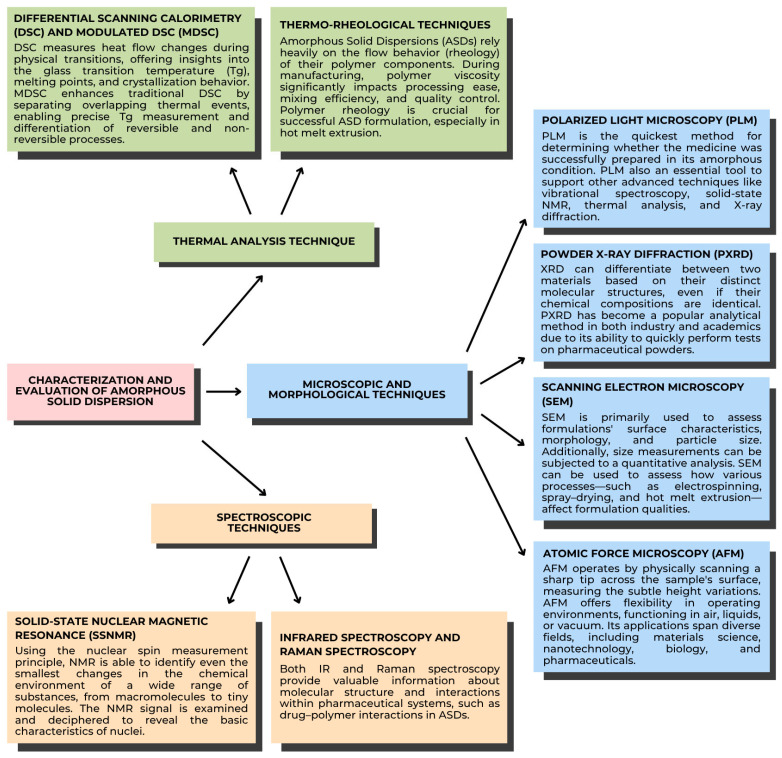
Characterization and evaluation of ASDs [72]. Adapted from [72], Elsevier, 2019.

The characterization of ASDs is essential for comprehending their stability. Various methods, including thermal analysis, microscopy, morphological assessment, and spectroscopy, are most effective for detailing properties like glass transition, molecular mobility, miscibility, crystallinity, and crystallization behavior. ASDs can be effectively characterized by thermal analysis, especially DSC and mDSC (Figure 4). Numerous thermal phenomena, including desorption, Tg, recrystallization, melting, and breakdown, can be detected using these methods. When it comes to correctly calculating Tg and separating overlapping events, mDSC is excellent. Analyzing ASDs is crucial because their mechanical properties reflect molecular-level interactions within the dispersion. One popular method for such evaluation is dynamic mechanical analysis (DMA) [65]. Amorphous solids exhibit characteristics of both solids and liquids, which vary with temperature. The physical stability of ASDs is directly connected to their molecular mobility. With greater internal energy, amorphous drugs exhibit enhanced solubility, leading to better dissolution and absorption. An ASD drug dissolves at the molecular level in a matrix, such as a polymer carrier, by definition. To assess the physical stability of the ASD during storage, evaluate its crystallinity [72]. Recrystallization of drugs from solid dispersions can reduce dissolution rates and subsequently negatively impact bioavailability; therefore, measurement techniques for detecting and characterizing crystallization events are of great importance [73].

### 5.1. Thermal Analysis Techniques

Thermal analysis is fundamental for evaluating the thermodynamic and kinetic properties of ASDs. Heat changes are measured using calorimetry. The temperature variations of the samples during particular processes, including physical transitions or chemical reactions, which can be either endothermic or exothermic, are correlated with these heat changes. The temperature profile can be programmed, and samples can be analyzed using current calorimetric equipment as a function of temperature or time. There are two kinds of calorimeters, based on their measuring principles: the heat compensation calorimeter, and the heat-exchanging calorimeter.

#### 5.1.1. Differential Scanning Calorimetry (DSC) and Modulated DSC (MDSC)

DSC evaluates thermal characteristics, specifically drug–natural polymer miscibility, physical stability, and glass transition temperature (Tg). While MDSC offers more precise Tg measurements by distinguishing between reversible and non-reversible heat flow, detecting enthalpy recovery, and phase separation, DSC assists in determining Tg and identifying thermal events like melting and crystallization. These methods are essential for assessing the performance and stability of ASDs, guaranteeing increased bioavailability and formulation efficacy. DSC measures heat flow changes during physical transitions, offering insights into the glass transition temperature (Tg), melting points, and crystallization behavior. MDSC enhances traditional DSC by separating overlapping thermal events, enabling precise Tg measurement and differentiation of reversible and non-reversible processes. For instance, MDSC has been used to study miscibility by identifying single Tg events in drug–natural polymer systems, which indicate homogeneity.

#### 5.1.2. Thermo-Rheological Techniques

ASDs rely heavily on the flow behavior (rheology) of their natural polymer components. During manufacturing, natural polymers’ viscosity significantly impacts their processing ease, mixing efficiency, and quality control. Natural polymers exhibit both elastic and viscous properties (viscoelasticity). Many pharmaceutical polymers are non-Newtonian, meaning that their viscosity changes with applied force. This process includes shear thinning and thickening. ASD formulations’ success depends on the natural polymers’ rheology, particularly in hot melt extrusion.

### 5.2. Microscopic and Morphological Techniques

Microscopy is used to characterize the solid state of ASDs. To investigate the physicochemical properties of ASDs, the pharmaceutical industry provides a range of microscopy techniques, such as glass transition, drug and natural polymer miscibility, crystallization behavior, crystallinity, morphology, heat events, and dissolution. Optical, electron, and scanning probe microscopy are the three general categories into which microscopic techniques fall. These include popular techniques that provide quick, non-destructive observations, such as atomic force microscopy, scanning electron microscopy, X-ray diffraction, and polarized light microscopy.

#### 5.2.1. Polarized Light Microscopy (PLM)

PLM studies the solid-state characteristics of these materials, specifically stability, miscibility, and crystallinity. With its high sensitivity, PLM is better than X-ray diffraction (XRD) at detecting small crystals and nuclei in ASDs. It helps with the tracking of ASDs’ physical stability and crystallization behavior over time.

#### 5.2.2. Powder X-Ray Diffraction (PXRD)

PXRD determines the crystalline components and evaluates the formulation’s crystallinity. PXRD is widely used because it can identify different crystal structures based on the arrangement of atoms in two materials, even if they have the same chemical composition. It offers a quantitative analysis of crystallinity, which is essential for determining how physically stable ASDs are under various storage circumstances.

#### 5.2.3. Scanning Electron Microscopy (SEM)

SEM examines the surface characteristics, morphology, and particle size of formulations. In order to visualize the onset of crystallization based on surface changes, SEM offers high-resolution images that complement information from methods such as X-ray diffraction (XRD). The impact of various manufacturing techniques (such as hot melt extrusion and spray-drying) on formulation properties is also investigated using this method. SEM can also be used in conjunction with energy-dispersive X-ray spectroscopy (EDS) to confirm the uniform distribution of drugs in natural polymer matrices and characterize drug–natural polymer interactions.

#### 5.2.4. Atomic Force Microscopy (AFM)

AFM provides high-resolution insights into structural and compositional heterogeneity by analyzing surface topography at the nanometer scale. The phase behavior, drug distribution, and miscibility of ASDs are all investigated with the aid of AFM. It is capable of differentiating between homogeneous and heterogeneous drug–polymer formulations and detecting phase separation. AFM is also used to quantify de-mixing through phase separation analysis and assess the stability of amorphous fractured films.

### 5.3. Spectroscopic Techniques

Spectroscopy plays a crucial role in understanding how drugs interact with natural polymers within ASDs. This technique relies on detecting changes at the molecular and atomic levels within the complex chemical environment of the ASD. By using various experimental setups, spectroscopy can provide insights at different scales, from the macroscopic to the nanoscopic. Spectroscopy is widely employed in the development and quality control of ASDs.

#### 5.3.1. Solid-State Nuclear Magnetic Resonance (ssNMR)

Solid-state NMR assesses drug–polymer interactions, miscibility, crystallinity, and molecular mobility. To evaluate stability and predict crystallization tendencies, ssNMR can quantify relaxation times. This study examines how polymers maintain pharmaceuticals in ASDs and investigates the impact of moisture on molecular interactions. Although ssNMR provides valuable quantitative and qualitative data, its widespread application is limited by its significant expense and prolonged data collection time.

#### 5.3.2. Infrared Spectroscopy and Raman Spectroscopy

Drug–polymer miscibility, phase separation, and molecular interactions in ASDs are examined using Raman and infrared (IR) spectroscopy. While Raman spectroscopy uses inelastic light scattering to detect interactions and phase separation, infrared spectroscopy uses vibrational energy absorption to study molecular structure and interactions. With small sample sizes, both methods offer quick, non-destructive analysis. Raman spectroscopy is excellent at identifying symmetric vibrations and nonpolar groups, whereas infrared spectroscopy is especially sensitive to polar groups. These techniques aid in formulation optimization by offering information on stability and drug–natural polymer interactions [72].

## 6. Development of Natural Polymer-Based ASD Systems

ASD is a proven way to boost the solubility of poorly water-soluble drugs. Instead of relying on synthetic carriers like PVP, recent research turns to natural polymers such as alginate, chitosan, pullulan, and plant proteins to create greener, biocompatible ASD matrices. These bio-based polymers can lock drugs in the high-energy amorphous state, prevent recrystallization, and control release. Table 3 highlights the current development of natural polymer-based ASDs as an attractive next step for both effective drug delivery and environmentally responsible formulation.

## 7. Solubility and Dissolution Studies of Natural Polymers

Enhancing the solubility of poorly water-soluble drugs remains a paramount challenge in pharmaceutical sciences, given its profound impact on bioavailability and therapeutic outcomes. Advanced formulation approaches leveraging polymeric carriers have emerged as promising strategies to address these limitations. Taokaew et al. [95] detected XRD, DSC, and aqueous dissolution studies that showed that the dissolution of nifedipine was improved by solid dispersions containing chitin microparticles as a naturally occurring polymeric carrier, rather than the pure drug. X-ray diffraction (XRD) and differential scanning calorimetry (DSC) analyses confirmed the amorphous state of nifedipine, with the absence of crystalline peaks and melting points. Aqueous dissolution studies showed enhanced drug release in gastric conditions (pH 1.2), achieving nearly 90–100% release over six hours. This improvement was attributed to the interaction between nifedipine and the chitin matrix, which prevented recrystallization and facilitated diffusion. The drug release followed a zero-order kinetics model with diffusion-based mechanisms, which demonstrated the potential of chitin as a natural polymer to enhance the bioavailability of poorly soluble drugs like nifedipine.

Panghal et al. [76] also stated that formulations with Modified Locust Bean Gum provide the best solubility and dissolution rate enhancement compared to pure atorvastatin. Enhanced equilibrium solubility was observed at a drug–polymer ratio of 1:6. The maximum dissolution rate was observed in the solid dispersion batch SD3 (i.e., 50% within 15 min), with maximum drug release after 2 h (80%), out of all solid dispersions. Formulations with Modified Locust Bean Gum, particularly at a 1:6 drug–polymer ratio (SD3), demonstrated the potential of this natural polymer to enhance the solubility and dissolution rate of poorly soluble drugs like atorvastatin.

Huang et al. [24] informed us that, by enhancing membrane permeability and dissolution behavior, chitosan oligosaccharides can increase the oral bioavailability of BCS-class IV pure drugs. Using curcumin (CUR) as a model, the researchers discovered that COS maintained supersaturation for 24 h and significantly accelerated dissolution rates in an ASD. Additionally, by opening intestinal tight junctions, COS improved membrane permeability, boosting curcumin absorption without sacrificing cell viability. In comparison to pure curcumin, pharmacokinetics studies showed that COS-ASD formulations enhanced bioavailability by 1.55 to 3.01 times. COS is a promising matrix for enhancing the therapeutic efficacy of poorly soluble medications, because the formulation maintained its physical stability for six months.

## 8. Drug Effectivity Studies of Natural Polymers

Enhancing bioavailability and therapeutic efficacy, especially in site-specific and systemic applications, depends critically on optimizing drug solubility. It can be inferred that improving the solubility, especially in the colon region, may aid in enhancing the bioavailability of Hes, as Rosiak et al. [87] identified the conditions in specific digestive system sections. Increasing the amount of dissolved hesperidin would enable intestinal bacteria to convert it into hesperetin, which could then be absorbed into systemic circulation and exert a pharmacological effect.

Shingel et al. [18] informed us that, in rat studies, the combination of letrozole with HA showed significantly higher plasma drug concentrations compared to pure drugs. The area under the curve (AUC) for letrozole increased by approximately 3-fold and 2.5-fold, respectively, with HA–drug formulations compared to pure drugs. In addition, Franca et al. [81] said that the DE90 values for crystalline and amorphous CTD under sink conditions were 32.58 ± 2.52% and 53.75 ± 2.62%, respectively. By reducing intermolecular attraction forces and promoting the average separation between molecules, changes to the drug’s solid state may enhance drug dissolution when compared to CTDa. This study demonstrated that by raising the plasma concentration and lowering intermolecular forces, the use of natural polymers or altering solid states can greatly improve drug bioavailability and dissolution.

Panghal et al. [76] found that the pharmacodynamic studies of the optimized solid dispersion (SD) batch SD3 showed significant improvements in efficacy at a dosage of 3 mg/kg/day in terms of lipid-lowering activity, including reductions in total cholesterol (94.59 ± 6.40 mg/dL), LDL (36.55 ± 6.24 mg/dL), and triglyceride levels (72.20 ± 4.21 mg/dL), and an increase in HDL cholesterol (58.45 ± 4.52 mg/dL), compared to the pure drug, which showed respective values of 125.80 ± 17.43 mg/dL, 56.09 ± 16.08 mg/dL, 88.20 ± 14.21 mg/dL, and 55.43 ± 7.19 mg/dL. This was ascribed to improved bioavailability brought about by the solid dispersion technique’s increased rate of dissolution. In addition to having a quicker onset of action, the SD3 formulation maintained its therapeutic effects for a longer period of time.

The TEL-loaded beads showed outstanding in vitro buoyancy, sustained drug release, and high entrapment efficiency, according to Uthumansha et al. [101]. TEL’s compatibility with the formulation ingredients was verified by IR spectroscopy, and the spherical shape of the beads was discovered by SEM analysis. Compared to the pure TEL suspension, the beads’ relative bioavailability was substantially higher (222.52%). Additionally, compared to the pure suspension, the formulation containing the natural polymer demonstrated a longer-lasting and more potent antihypertensive effect.

## 9. Stability Studies of Natural Polymers

Attaining and preserving stability in pharmaceutical formulations is crucial for ensuring consistent efficacy, shelf-life, and patient safety. Advanced carrier systems have shown significant potential in maintaining active pharmaceutical ingredients (APIs) under diverse circumstances. Guan et al. [25] reported that the physical stability of the solid dispersion was assessed over a period of 10 days at 60 °C, 92.5% humidity, and 4500 ± 500 lx lighting; the alginate-based solid dispersion exhibited commendable stability under accelerated conditions. Medication dissolution rates showed only slight declines. While Raman mapping and scanning electron microscopy (SEM) studies showed limited phase separation, which was attributed to water plasticization in high-humidity conditions, X-ray powder diffraction (XRPD) confirmed very little recrystallization of the drug. The disintegration rate was largely unaffected by this minor structural change. According to Guan et al. [25], solid dispersions based on alginate demonstrated good physical stability under accelerated conditions, with only minor drops in dissolution rates and little drug recrystallization.

After 180 days of accelerated stability testing, Singh et al. [102] reported that the MLBG dispersions showed good thermal stability and retained their amorphous nature. The results showed that MLBG dispersions had good thermal stability, remaining amorphous throughout the testing period. For consistent drug release profiles and long-term efficacy, this stability is essential. Over 180 days, Singh et al. discovered that MLBG dispersions stayed stable and amorphous, but POLO-based formulations displayed decreased crystallinity, potentially impairing their performance.

According to 39 studies on physical stability, solid dispersions using sodium alginate as a carrier maintained their stability for 90 days at room temperature, according to Borba et al. [100]. This suggests that sodium alginate works as an efficient anti-plasticizer that prevents drug recrystallization. Over the course of 90 days at room temperature, 39 SD formulations were investigated. According to the results, the SDs remained stable during the testing period, retaining their original properties without experiencing any notable changes to their amorphous form or drug dissolution capabilities.

## 10. Critical Evaluation and Mechanistic Insights

ASDs represent an effective strategy to address common pharmaceutical challenges, particularly the low solubility and poor bioavailability of active pharmaceutical ingredients (APIs). The lack of stability in drugs, particularly those in crystalline form, severely limits their therapeutic efficacy and bioavailability. This instability results in reduced solubility, slower dissolution rates, and diminished absorption, which together hinder the drug’s potential for reaching the target site in sufficient concentrations. To address these issues, the development of ASDs, where the drug is dispersed in an amorphous form within a polymeric matrix, has emerged as a promising approach [60]. By preventing recrystallization, ASDs enhance solubility and dissolution rates, ultimately improving the bioavailability and pharmacological efficacy of poorly soluble drugs [65].

**Figure 5:**
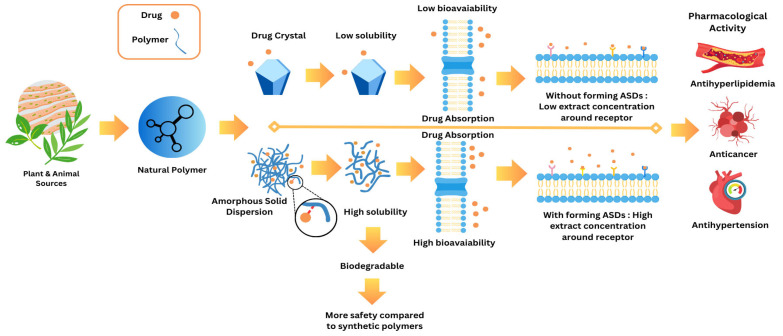
Drug–natural polymer absorption pathways.

The principal mechanism by which ASDs enhance drug solubility lies in the transformation of the drug from a crystalline to an amorphous state and its homogeneous dispersion within a polymer matrix (Figure 5). Unlike their crystalline counterparts, amorphous compounds possess higher internal energy and lack the long-range molecular order characteristic of crystalline lattices. This disordered structure leads to increased free volume and greater molecular mobility, which collectively reduce the energy barrier for dissolution and facilitate faster drug release in aqueous environments. Consequently, ASDs significantly improve the apparent solubility and dissolution rate of poorly water-soluble drugs, particularly those classified under BCS class II and IV, enabling superior absorption profiles and enhanced oral bioavailability [16,103].

Beyond solubility enhancement, ASDs also play a critical role in improving the physical stability of the drug by preventing its recrystallization—a major limitation in amorphous formulations when left unprotected. The polymer matrix acts not merely as a carrier but as a stabilizing medium that restricts the molecular mobility of the dispersed drug through intermolecular interactions such as hydrogen bonding, van der Waals forces, or ionic interactions. These interactions reduce the thermodynamic drive toward crystallization by disrupting the nucleation and crystal growth processes. Moreover, polymers with a high glass transition temperature (Tg) elevate the overall Tg of the dispersion system, thereby immobilizing the drug molecules and kinetically hindering phase separation or recrystallization during storage. This stabilization mechanism is particularly crucial under conditions of thermal and humidity stress, where amorphous drugs are otherwise prone to transition back into their crystalline form. Therefore, the ASD strategy provides a dual benefit: it enhances solubility through increased molecular dispersion and maintains stability by kinetically and thermodynamically suppressing recrystallization, collectively ensuring a robust and bioavailable formulation suitable for long-term pharmaceutical application. Natural polymers, such as chitosan, alginate, and pectin, have gained increasing attention in the formulation of ASDs due to their biocompatibility, biodegradability, and sustainability. Unlike synthetic polymers, natural polymers offer several advantages, including lower toxicity, safer degradation byproducts, and renewable sources [104]. They can stabilize the amorphous drug state by forming hydrogen bonds with drug molecules, preventing recrystallization, and improving the solubility and stability of the drug [19]. For instance, chitosan, a polysaccharide derived from chitin, not only stabilizes drugs in their amorphous form but also enhances drug permeability by interacting with mucus layers, improving drug absorption. Similarly, alginate and pectin, both derived from plant sources, have demonstrated the ability to enhance drugs’ solubility and stability [105]. The use of these natural polymers ensures a more sustainable and environmentally friendly approach to drug formulation, which aligns with the increasing demand for green pharmaceutical technologies.

In comparison to crystalline drug forms, which dissolve slowly due to tightly packed lattice structures, ASDs using natural polymers significantly improve the dissolution rate. The absence of a crystalline structure in amorphous drugs reduces the energy required for dissolution, thus increasing the rate at which the drug enters the bloodstream. This rapid dissolution is particularly beneficial for drugs targeting specific receptors or tissues, as it allows for higher concentrations of the drug to reach the site of action more efficiently. The enhanced solubility of the drug also facilitates its absorption through biological membranes, ensuring that the therapeutic dose reaches the target site effectively.

Additionally, the use of natural polymers in ASD formulations improves the therapeutic efficacy of the drug by increasing the concentration of the drug available at the receptor sites. The increased solubility and faster dissolution rates allow the drug to interact with its target receptor in higher concentrations, thereby enhancing its pharmacological activity. This mechanism is especially critical for the treatment of chronic conditions such as cancer and cardiovascular diseases, where sustained therapeutic levels are required for optimal drug efficacy.

Natural polymers offer distinct advantages over synthetic ones in drug formulations. They not only enhance the solubility, dissolution rate, and bioavailability of poorly soluble drugs but also provide biocompatibility and sustainability. The biodegradability of natural polymers reduces the environmental impact [106], while their ability to stabilize the amorphous state of the drug contributes to the overall effectiveness of the ASD system. By offering a more environmentally friendly and safer alternative, natural polymers are well positioned to meet the growing demand for innovative and sustainable pharmaceutical solutions [19].

From the authors’ perspective, the development of ASD systems using natural polymers represents a significant advancement in the field of pharmaceutical formulations. The unique properties of natural polymers, such as their ability to stabilize amorphous drugs, enhance solubility, and improve bioavailability, offer clear advantages over synthetic polymers. This approach aligns with the growing trend towards sustainable pharmaceutical manufacturing and provides a promising solution to the challenges posed by poorly soluble drugs.

## 11. Challenges and Limitations of Natural Polymer-Based Amorphous Solid Dispersions (NP-ASDs)

Despite the growing interest in natural polymer-based amorphous solid dispersion (NP-ASD) systems as biocompatible and sustainable alternatives to synthetic carriers, their translational potential is still hindered by several complex scientific, technical, and regulatory challenges. A critical understanding of these limitations is necessary not only to guide future research but also to inform realistic expectations for clinical and industrial application. These challenges span from inherent variability in natural polymers and difficulties in molecular-level characterization to processing constraints, stability limitations, and regulatory hurdles (Table 4). Each of these factors introduces uncertainty in formulation performance, scalability, and long-term efficacy.

### 11.1. Variability and Inconsistency of Natural Polymers

One of the foremost limitations of NP-ASDs lies in the intrinsic batch-to-batch variability of natural polymers. Unlike synthetic polymers, which can be produced under tightly controlled monomer composition and molecular weight distribution, natural polymers are extracted from biological sources such as plants, marine organisms, or animals. As a result, their physicochemical properties—such as molecular weight, degree of substitution, branching, and purity—are often influenced by factors like seasonality, extraction methods, geographical origin, and species variation. This compositional heterogeneity can severely impact critical parameters, including drug–polymer miscibility, recrystallization inhibition capacity, and physical stability of the ASD matrix. For instance, alginates sourced from different seaweeds may vary in their mannuronic/guluronic acid ratio, which directly affects their gelation behavior and drug interaction potential. Such variability complicates quality assurance, scalability, and regulatory compliance, especially under Good Manufacturing Practice (GMP) conditions.

### 11.2. Complexity in Predicting Drug–Polymer Interactions

NP-ASDs rely on strong intermolecular interactions—primarily hydrogen bonding, ionic interactions, and van der Waals forces—between the amorphous drug and the natural polymer matrix to stabilize the high-energy amorphous state. However, predicting and characterizing these interactions at the molecular level remains a formidable challenge. Techniques such as Fourier-transform infrared (FTIR) spectroscopy, solid-state nuclear magnetic resonance (ssNMR), and molecular dynamics simulations can provide insights into drug–polymer binding, yet their routine application is limited due to technical complexity, cost, and accessibility. Furthermore, the amorphous nature of both the drug and the polymer introduces kinetic instability and potential for phase separation, particularly when the drug loading approaches the miscibility limit. This complexity limits the ability to rationally design NP-ASDs with predictable physical stability and dissolution performance.

### 11.3. Formulation and Processing Constraints

Natural polymers often exhibit poor thermal stability, sensitivity to moisture, and limited solubility in common pharmaceutical solvents. These characteristics restrict the choice of processing methods and reduce the formulation flexibility. For instance, while hot melt extrusion (HME) is a widely adopted technique for producing ASDs with synthetic polymers, many natural polymers degrade or denature at the high temperatures required, making them unsuitable for thermal processing. Similarly, solvent-based methods such as spray-drying or solvent evaporation may be constrained by the insolubility of certain natural polymers in pharmaceutically acceptable solvents, or by the high viscosity of polymer solutions, which can affect atomization and film formation. Additionally, the presence of moisture in hygroscopic natural polymers like chitosan or pectin can promote hydrolytic degradation or plasticization, further compromising stability during processing or storage.

### 11.4. Limited Shelf-Life and Stability Under Stress Conditions

A central limitation of NP-ASDs is the difficulty in maintaining the amorphous state of the drug under long-term or accelerated stability conditions. Natural polymers often exhibit lower glass transition temperatures (Tg) compared to synthetic polymers, resulting in higher molecular mobility and a greater risk of drug recrystallization. Under high humidity and temperature, water uptake by hydrophilic natural polymers can act as a plasticizer, reducing Tg and promoting phase separation or crystallization of the drug. Although some natural polymers like alginate and chitosan have demonstrated promising stability in short-term studies, comprehensive long-term evaluations under ICH storage conditions remain sparse. Without robust stabilization, even minor physical transformations can lead to dramatic decreases in dissolution rates and bioavailability, undermining the therapeutic advantage of the ASD system.

### 11.5. Immunogenicity and Allergenicity Risks

Natural polymers derived from biological sources inherently carry the risk of immunogenic or allergic reactions, particularly when used in parenteral or mucosal delivery. For example, chitosan derived from crustacean shells may contain residual proteins or endotoxins that could elicit immune responses. Similarly, protein-based carriers such as gelatin or albumin must be rigorously purified and tested for viral contaminants and antigenicity. These risks demand comprehensive toxicological evaluation and may limit the use of certain polymers in specific patient populations (e.g., pediatric, immunocompromised). Moreover, regulatory bodies require extensive documentation to prove safety and biocompatibility, especially when polymers are sourced from animals or genetically variable plant species.

### 11.6. Analytical and Regulatory Limitations

The analytical characterization of NP-ASDs is inherently more complex due to the heterogeneous, amorphous nature of the matrix and the overlapping spectroscopic signatures of natural polymers and drugs. Quantifying crystallinity, monitoring drug–polymer miscibility, and detecting early-stage recrystallization require advanced, often orthogonal techniques such as PXRD, DSC, ssNMR, and AFM. These are not always feasible in industrial settings, due to resource and time constraints. From a regulatory standpoint, the lack of standardized monographs or specifications for many natural polymers (e.g., microbial-derived pullulan, plant exudates) poses challenges for product registration. Ensuring batch uniformity, reproducibility, and safety under global regulatory frameworks remains a major bottleneck for commercialization.

## 12. Conclusions and Future Perspectives

### 12.1. Conclusions

Natural polymer-based ASD systems represent a transformative advancement in pharmaceutical formulation science, offering a strategic solution to overcome the limitations of poorly water-soluble drugs. This review has highlighted the unique capabilities of natural polymers—such as chitosan, alginate, pectin, hyaluronic acid, and cyclodextrins—in stabilizing amorphous forms, improving solubility and dissolution, and enhancing bioavailability. These polymers not only engage in favorable molecular interactions to inhibit recrystallization and sustain supersaturation but also possess superior safety, biocompatibility, and biodegradability profiles when compared to their synthetic counterparts. The collective evidence from recent studies demonstrates that natural polymers can serve as both functional and biologically harmonious carriers in ASD systems, achieving therapeutic effectiveness while minimizing toxicity risks. Ultimately, this review underscores the valuable role of natural polymers in redefining the design of safer, more efficient drug delivery systems, affirming their potential as the future standard in amorphous formulation technology.

### 12.2. Future Perspectives

Looking ahead, natural polymer-based ASDs are poised to become a central platform in the development of next-generation drug delivery systems, not only in the oral route but also in targeted, mucosal, transdermal, and implantable applications. Their inherent versatility and compatibility with biological systems position them as ideal candidates for emerging areas such as personalized medicine, pediatric therapeutics, cancer therapy, and biopharmaceutical stabilization. With the increasing demand for eco-friendly, patient-safe, and high-performance drug carriers, natural polymers offer a sustainable and clinically promising alternative to synthetic materials.

However, to fully unlock the potential of natural polymers in ASD systems, several scientific gaps must be addressed through future research. First, a deeper understanding of drug–polymer molecular interactions is essential—this includes the use of advanced spectroscopic and simulation-based techniques (e.g., solid-state NMR, FTIR mapping, molecular dynamics) to predict and validate stabilizing forces such as hydrogen bonding, hydrophobic stacking, or electrostatic interactions. Second, the mechanistic role of natural polymers in inhibiting nucleation and crystal growth must be elucidated under both in vitro and in vivo conditions, particularly under gastrointestinal stress and storage scenarios. Third, future work should also explore the development of hybrid or synergistic polymer systems, such as combining two or more natural polymers or integrating natural and semi-synthetic polymers to achieve optimal performance in terms of solubility, dissolution control, and mechanical properties.

In addition, scaling up production and ensuring regulatory compliance will require further formulation and process engineering studies. Techniques such as hot melt extrusion, spray-drying, and solvent-free methods must be optimized specifically for natural polymers, taking into account their sensitivity to temperature and moisture. Finally, in vivo pharmacokinetic, pharmacodynamic, and safety profiling of natural polymer-based ASDs must be expanded through preclinical and clinical investigations to confirm long-term biocompatibility and therapeutic effectiveness across various drug classes and patient populations.

In conclusion, while significant progress has been made, the journey toward fully integrating natural polymers into mainstream ASD platforms requires continued interdisciplinary research. By advancing both mechanistic understanding and translational development, researchers can harness the full potential of natural polymers to revolutionize drug solubility enhancement in a safe, effective, and sustainable manner.

## Figures and Tables

**Table 1:** Natural polymers used in ASDs.

No	Natural Polymer	Structure (Monomer)	Sources	Function	Ref.
1.	Albumin	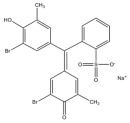	Human serum, plants (such as peanuts, sunflowers, passion fruit, etc.), and animals (such as cows, chickens, salmon, etc.)	Albumin acts as a drug reservoir that can improve the bioavailability and biodistribution of drugs.	[28]
2.	Alginate	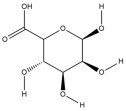	Brown seaweed	Alginate has been used as tablet disintegrants, both in the more hydrophobic tablet formulations and in difficult hard tablets with a binder; calcium alginate could offer mediocre disintegration performance.	[29]
3.	Cellulose	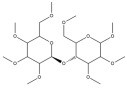	Cell walls of plants	Cellulose acts as a binder and diluent in formulations for oral tablets and capsules that use both direct compression and wet granulation techniques. Additionally, it has certain disintegrant and lubricating qualities that are helpful for direct tableting.	[30]
4.	Chitin	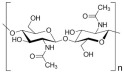	Exoskeletons of shellfish and insects (crustacean shells)	Chitin’s great water absorption capacity and ability to increase porosity have made it widely recognized as an effective tablet disintegrant.	[31]
5.	Chitosan	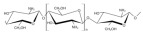	Deacetylation of chitin	Chitosan serves in prolonging the duration of preprogrammed drug delivery, enabling stimuli-responsive smart delivery to target sites, protecting encapsulated drugs within the mesh network from adverse environments, and facilitating mucoadhesion and penetration through cell membranes.	[32]
6.	Cyclodextrin	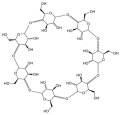	Potatoes, corn, maize, and cassava starches	Cyclodextrin functions to reduce or eliminate unpleasant taste and smell, improving the aqueous solubility, dissolution, and bioavailability of drugs, minimizing adverse drug reactions like gastrointestinal and ocular irritation, and turning liquid drugs into microcrystalline or amorphous powders.	[33]
7.	Gelatin	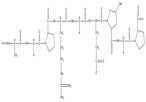	Hydrolysis of collagen, which is extracted from the skin, connective tissues, and bones of animals	Gelatin is utilized as a thickening agent, stabilizer, and emulsifier. It has been found in drug delivery due to its ability to produce hydrogels.	[34]
8.	Guar gum	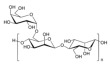	Seeds of *Cyamopsis tetragonolobus* of the Leguminosae family	Guar gum is feasible to create forms like films and gels, as well as high-viscosity solutions with a high ability to retain water, used as a stabilizer, thickening, emulsifying agent, suspending agent, and viscosity enhancer.	[35]
9.	Gum acacia	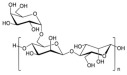	Exudate gum obtained from *Acacia senegal*	Acacia gum acts as a thickening, stabilizing, emulsifying, and microencapsulating agent.	[36]
10.	Hyaluronic acid	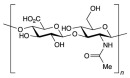	Animal tissues	Hyaluronic acid is utilized as a sustained-release drug carrier, which has a long-acting impact and can delay drug release.	[37]
11.	Kappa- carrageenan	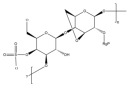	Red edible seaweeds	Carrageenan is an innovative extrusion aid for pellet production. It is also employed as a gelling agent and a viscosity enhancer to achieve controlled drug release and extended retention.	[38]
12.	Locust bean gum	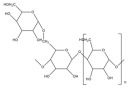	Grinding the endosperm of beans or brown pods.	Locust bean gum functions as a matrix-forming material in tablets.	[39]
13.	Pectin	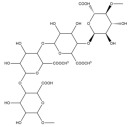	Cell walls of terrestrial plants	Pectin functions as a binder and disintegrant in pharmaceutical tablets to ensure proper drug absorption and effectiveness.	[40]
14.	Starch	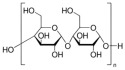	Grains, cereals, and potatoes	Starch functions as a carrier, effectively modulating the release of active substances, and enhances the innate physiological activity of different active components.	[41]
15.	Xanthan gum	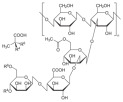	Microorganism *Xanthomonas campestris* of the Xanthomonadaceae family	Xanthan gum plays an important role in food and pharmaceutical applications as binder, thickener, and emulsion stabilizer.	[42]
16.	Xyloglucan	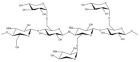	Plant cell walls	Xyloglucan acts as a stabilizer, solubilizer, thickener, gelling agent, and binder in the food and pharmaceutical industries.	[43]

**Table 2:** The advantages of natural polymers as compared to non-natural polymers.

No	Aspect	Natural Polymers	Synthetic Polymers	Ref.
1.	Source	Derived from plants, animals, and microorganisms (e.g., chitosan from shrimp shells, gelatin from animal collagen, alginate from seaweed).	Derived from petrochemical products or synthesized in laboratories.	[15,55]
2	Biodegradability	Generally biodegradable and eco-friendly.	Often non-biodegradable and environmentally persistent.	[44,48,54]
3	Biocompatibility and Safety	Safer, low toxicity, and rarely cause allergic or toxic reactions.	May cause adverse effects (e.g., PVP linked to tumor formation in implants).	[15,48,49]
4	Biological Activity	Naturally bioactive, supporting cell interaction and tissue regeneration.	Typically inert unless chemically modified.	[21,22,48,52]
5	Safe Degradation Products	Degrade without leaving harmful residues.	May produce toxic byproducts or environmental pollutants.	[44,53,54]
6	Sustainability and Availability	Abundant and renewable, especially from marine sources.	Limited by petrochemical availability.	[27,54]
7	Mechanical and Functional Properties	Often possess properties such as mucoadhesion, prolonged drug release, high water absorption, and enhanced bioavailability.	Generally superior in mechanical strength and stability, but not always multifunctional.	[22,48,52]

**Table 3:** Current development of natural polymer-based ASD systems.

Drug	Pure Drug Solubility	Natural Polymer (Class)	Bond Reaction Mechanism	ASD System Solubility, Dissolution Rate, and Release Behavior	In Vitro and In Vivo Results	Stability	Ref.
Amphotericin	1 µg/mL in water	Cyclodextrin	The ASD formulations of Amphotericin B (AmB) involve hydrogen bonding with cyclodextrin polymers, van der Waals and dipole–dipole interactions, and host–guest inclusion complexation with cyclodextrin cavities.	The solubility of pure AmB in water is about 1 µg/mL, but when formulated as ASD using poly-γ-CD, it increased by a factor of 127, reaching 761 µg/mL. Additionally, dissolution tests showed that only 60% of pure AmB dissolved in 45 min at pH 7.4	-	The ASD formulation maintains its amorphous state due to strong interactions between amphotericin B (AmB) and cyclodextrin polymers, preventing crystallization. Additionally, the thermal analysis confirms that the ASD remains stable at elevated temperatures.	[74]
Atovarstatin	Aqueous solution at pH 2.1 is about 0.0204 mg/mL [75]	Sodium alginate	The analysis suggests a possible hydrogen bond interaction between the drug (hydroxyl (-OH), amine (-NH)) and sodium alginate (SA) (carboxylate (-COO^−^)) in the ASD system.	The ASD formulation enhanced drug dissolution and supersaturation maintenance, with an AUC_0–480_ of 226.8 ± 10.3 mg·min/mL, compared to 109.2 ± 1.4 mg·min/mL for crystalline CTD under non-sink conditions.	-	-	[76]
Azithromycin	Poor aqueous solubility (~0.1 mg/mL) [77]	Chitosan, alginate	The analysis suggests hydrogen bond formation between the carboxyl (COOH) and amine (-NH_2_) groups of azithromycin and the amide (-CONH_2_) or ester (-COO) groups of the polymer.	Azithromycin combined with chitosan and alginate increased the rate of azithromycin dissolution when compared to the pure azithromycin; after 5 min, the drug release from the solid dispersion was 95%, but only 10% of pure azithromycin was released.	-	-	[78]
Bexarotene	Poor fluid solvency, very poor solubility of 0.00018 ± 0.0002 mg/mL in distilled water.	Modified Locust Bean Gum (MLBG)	The major change occurred in the peak of BEX-MLBG solid dispersion at a wavelength of 3302 cm^−1^ for -OH stretching, which might be due to inter- and intramolecular hydrogen bonding of MLBG.	The drug exhibited extremely poor solubility in water (0.00018 mg/mL), its but after ASD preparation, solubility increased to 0.00340 mg/mL (bexarotene–Modified Locust Bean Gum 1:4), showing an approximately 18–19-fold enhancement.	-	The Modified Locust Bean Gum dispersions exhibited good thermal stability and maintained their amorphous nature over 180 days of accelerated stability testing.	[79]
Chlortalidone	A low aqueous solubility of 0.191 mg/mL in water [80]	Sodium alginate	The analysis suggests a possible hydrogen bond interaction between the drug (hydroxyl (-OH), amine (-NH)) and sodium alginate (SA) (carboxylate (-COO^−^)) in the ASD system.	The ASD formulation enhanced drug dissolution and supersaturation maintenance, with an AUC_0–480_ of 226.8 ± 10.3 mg·min/mL, compared to 109.2 ± 1.4 mg·min/mL for crystalline CTD under non-sink conditions.	-	-	[81]
Curcumin (CUR)	Poor water solubility (0.6 µg/mL in water)	Chitosan oligosaccharide (COS)	FTIR analysis showed the disappearance of the O–H stretching peak of curcumin at 3514 cm^−1^ and a reduction in the C=O stretching of chitosan oligosaccharide at 1608 cm^−1^, indicating hydrogen bond formation between the hydroxyl groups of curcumin and functional groups of chitosan oligosaccharide.	The dissolution of pure curcumin (60.62 μg/mL) significantly improved after forming an ASD with chitosan oligosaccharide, reaching 97.85–101.21 μg/mL and maintaining supersaturation for 24 h.	-	The curcumin–chitosan oligosaccharide ASD showed at least six months of physical stability in the XRPD patterns. The results show that a stable amorphous form of curcumin is produced by the co-milling process with chitosan oligosaccharide as the matrix.	[24]
Etoricoxib	24.49 µg/mL pure etoricoxib in distilled water [82]	Xanthan gum, guar gum, and gum acacia	-	Increased solubility, as evidenced by an amorphous content of 98.2 ± 1.3%, which was markedly higher compared to other formulations.	-	-	[83]
Famotidine	0.1% (*w*/*v*) at 293 K [84]	Xyloglucan	-	Enhanced the solubility of the drug with the optimal drug/xyloglucan ratio (1:1), with a solubility value of 10.436 ± 0.045 mg/mL mg/mL, compared to the much lower solubility of pure famotidine (0.405 ± 0.002 mg/mL).	-	-	[85]
Hesperidin	4.95 µg·mL^−1^ [86]	Sodium alginate (Soluplus)	-	Enhanced, wherein Hes/Sol 1:5 *w*/*w* has the best solubility (about 300-fold in each medium), with a maximum of 2.710 ± 0.004 mg·mL^−1^ for the Hes/Sol system.	-	Stability studies indicate that the strength of these bonds is insufficient to maintain the amorphous state of Hes under stress conditions (25 °C and 60 °C 76.4% RH).	[87]
Ibuprofen	21 µg/mL of pure ibuprofen was soluble in distilled water	Xanthan gum, guar gum, and gum acacia	-	Pure ibuprofen had a solubility of 21 µg/mL in distilled water, whereas the optimized ASD formulation (IB11) increased solubility to 115 µg/mL, approximately 5.5 times higher. Similarly, dissolution tests revealed that while the pure drug had a limited release over 90 min, the ASD formulation achieved 97.2% drug release in the same period.	-	-	[88]
Indomethacin	Very poor aqueous solubility (0.937 μg/mL) [89]	Alginate	The analysis suggests an interaction between the tertiary ammonium (-NR_3_^+^) of indomethacin and the carboxyl (-COO^−^) group of alginates.	Alginate considerably increased the rate of indomethacin dissolution when compared to the pure drug; after five minutes, the drug release from the solid dispersion was nearly total, but only 5.3% of indomethacin was released from the pure drug.	-	Alginate-based solid dispersion presented good stability under accelerated conditions after 10 days when examined at 60 °C and 92.5% humidity.	[25]
Irinotecan	Poorly water-soluble, 20 micrograms/mL [90]	Hyaluronic acid	-	HA–irinotecan and pure drug were administered as suspensions in corn oil. HA–irinotecan and pure drug were suspended in corn oil to give the drug concentration of 13.3 mg/mL (133 mg/mL of HA–irinotecan).	Irinotecan and its active metabolite, SN-38, were eliminated from rat plasma within 48 h after administration. The area under the curve (AUC) and maximum plasma concentration (Cmax) of irinotecan were higher when administered in pure form compared to the HA–irinotecan formulation at a dose of 50 mg/kg.	The process of preparing HA–drug complexes through solvent evaporation and 10–20 cycles of extrusion did not affect the contents or purity of the drug components in the solid dispersions. The purity of the drugs in the HA–drug formulations remained high (irinotecan: 98.6%).	[18]
Letrozole	Poorly water-soluble, 0.02–0.05 mg/mL [91]	Hyaluronic acid	-	Pure letrozole was suspended in water and administered as aqueous drug suspensions with drug concentrations of 2.5 mg/mL and 0.3 mg/mL.	Combination of letrozole with HA showed significantly higher plasma drug concentrations compared to the pure drug. The area under the curve (AUC) for letrozole increased by approximately 3-fold and 2.5-fold, respectively, with HA–drug formulations compared to the pure drug.	The process of preparing HA–drug complexes through solvent evaporation and 10–20 cycles of extrusion did not affect the content or purity of the drug components in the solid dispersions. The purity of the drugs in the HA–drug formulations remained high (letrozole: >99.0%).	[18]
Lovastatin	The solubility of lovastatin in water at 25 °C is 0.84 mg/mL [92]	Alginate	-	Alginate considerably increased the rate of lovastatin dissolution when compared to the pure drug; after 30 min, the drug release from the solid dispersion was 79.9%, but under 30% of lovastatin was released from the pure drug.	-	Alginate-based solid dispersion presented good stability under accelerated conditions after 10 days when examined at 60 °C and 92.5% humidity.	[25]
Naproxen	Very poor water solubility (0.025 mg/mL at 25 °C)	Chitosan	-	When chitosan was particularly synthesized through co-grinding at a 1:9 drug-to-polymer ratio, markedly enhanced solubility (up to 17-fold) and accelerated dissolution rate (exceeding 10 times) were observed, leading to expedited and more comprehensive drug release.	-	-	[93]
Nifedipine	Insoluble in water, 4–5 μg·mL^−1^ [94]	Chitin	The analysis suggests an interaction between the amide (NH) of chitin and the carbonyl (C=O) of nifedipine, as evidenced by the observed peak shifts.	The dissolution of nifedipine was improved by solid dispersions containing chitin, as opposed to the pure drug. The drug could release at roughly 90%, which was higher than the release in the neutral-pH medium (60%) over six hours.	-	The ASD of nifedipine showed stability at 30 °C and 75% relative humidity (RH). After two and four weeks of storage, the X-ray diffraction (XRD) patterns of pure nifedipine revealed no changes, indicating that its crystalline structure remained intact.	[95]
Oxymatrine	Highly water-soluble (~100 g/L)	Alginate–chitosan floating beads	-	Pure oxymatrine (OM) in alginate–chitosan beads showed poor encapsulation (38.92%) and rapid release (1.5 h), while its solid dispersion with ethyl cellulose improved encapsulation (67.07%) and sustained release (12 h), enhancing solubility and control.	-	-	[96]
Prednisolone	Poorly soluble in water: 0.133 mg/mL at 25 °C [97]	Albumin	-	Formulating PRD in BSA solid dispersions allowed an instant dissolution, with almost 90% dissolved within the first 10 min. PRD release rates from BSA complex were comparable to those obtained from PRD aqueous solution (*p* > 0.05), with approximately 50% of PRD diffused to the receptor compartment after 6 h.	-	Bovine serum albumin (BSA) has been widely used in numerous pharmaceutical and medical applications thanks to its stability, biocompatibility and low immunogenicity. The two drying techniques used to form the solid dispersions had no impact on BSA stability.	[98]
Telmisartan	Poorly water-soluble drugs, practically insoluble at pH 3–7 (0.09 μg/mL in water) [99]	Sodium alginate	The analysis suggests intermolecular hydrogen bond interactions between the carbonyl (-C=O) group of telmisartan (TEL) and the hydroxyl (-OH) group of sodium alginate (SA)	The SD of telmisartan achieved improvements ranging from 12.6- to 16.7-fold in the TEL’s apparent aqueous 252 solubility (0.5 µg/mL at 25 °C).	-	According to the 39 physical stability investigations, SD utilizing sodium alginate as a carrier stayed constant over 90 days at room temperature, indicating that sodium alginate functions as an effective anti-plasticizer agent that inhibits drug recrystallization.	[100]
		Sodium alginate beads	-	Oil-entrapped beads had a prolonged drug release over 12 h in the simulated gastric fluid, and the cumulative release was 92.68 ± 3.09%.	In vivo experiments demonstrate that the gastro-retentive properties of alginate beads effectively regulate, enhance, and prolong the systemic absorption of telmisartan (TEL). The results confirm that TEL maintains its antihypertensive activity and significantly improves therapeutic efficacy.	-	[101]

**Table 4:** Summary of key challenges and limitations in the development of natural polymer-based amorphous solid dispersions (NP-ASDs), their underlying causes, and potential implications for formulation performance.

Challenge Category	Specific Limitations	Implications/Consequences
Physicochemical Variability	- Batch-to-batch inconsistency; variable molecular weight, purity, and composition	- Unpredictable performance; difficult formulation reproducibility
Interaction Complexity	- Uncertain hydrogen bonding; difficult to model drug–polymer affinity	- Low miscibility; risk of phase separation or recrystallization
Processing Limitations	- Low thermal stability; solvent incompatibility; high viscosity	- Incompatible with HME or spray-drying; formulation instability
Stability Challenges	- Moisture uptake; low Tg of natural polymers; plasticization risk	- Crystallization during storage; reduced shelf-life and dissolution performance
Immunogenicity Risks	- Source from animal/marine origin; residual proteins/endotoxins	- Allergenicity; regulatory limitations for sensitive populations
Analytical Difficulties	- Overlapping FTIR/NMR signals; amorphous heterogeneity	- Complex characterization; delayed formulation optimization
Regulatory Barriers	- Lack of pharmacopeial standards; safety/toxicity profile incomplete	- Hurdles for global approval; need for extensive safety documentation

## Data Availability

No new data were created or analyzed in this study. Data sharing is not applicable to this article.

## Abbreviations

The following abbreviations are used in this manuscript:

ASD	Amorphous solid dispersion
API	Active pharmaceutical ingredient
PVP	Polyvinylpyrrolidone
HPMC	Hydroxypropyl methylcellulose
NP-ASDs	Natural polymer–based ASDs
PEG	Polyethylene glycol
DNA	Deoxyribonucleic acid
RNA	Ribonucleic acid
HME	Hot melt extrusion
DMA	Dynamic mechanical analysis
DSC	Differential scanning calorimetry
PLM	Polarized light microscopy
PXRD	Powder X-ray diffraction
SEM	Scanning electron microscopy
AFM	Atomic force microscopy
ssNMR	Solid-state nuclear magnetic resonance
AUC	Area under the curve
